# Prediction Based Proactive Thermal Virtual Machine Scheduling in Green Clouds

**DOI:** 10.1155/2014/208983

**Published:** 2014-03-11

**Authors:** Supriya Kinger, Rajesh Kumar, Anju Sharma

**Affiliations:** ^1^Department of Computer Science and Engineering, SGGS World University, Fatehgarh Sahib, Punjab, India; ^2^School of Mathematics and Computer Applications, Thapar University, Patiala, India

## Abstract

Cloud computing has rapidly emerged as a widely accepted computing paradigm, but the research on Cloud computing is still at an early stage. Cloud computing provides many advanced features but it still has some shortcomings such as relatively high operating cost and environmental hazards like increasing carbon footprints. These hazards can be reduced up to some extent by efficient scheduling of Cloud resources. Working temperature on which a machine is currently running can be taken as a criterion for Virtual Machine (VM) scheduling. This paper proposes a new proactive technique that considers current and maximum threshold temperature of Server Machines (SMs) before making scheduling decisions with the help of a temperature predictor, so that maximum temperature is never reached. Different workload scenarios have been taken into consideration. The results obtained show that the proposed system is better than existing systems of VM scheduling, which does not consider current temperature of nodes before making scheduling decisions. Thus, a reduction in need of cooling systems for a Cloud environment has been obtained and validated.

## 1. Introduction

Cloud computing is a rapidly emerging technology that uses the internet and central remote servers to maintain the data and applications [1]. There is increasing demand for computational power by scientific, business, and web applications which has led to creation of large scale data centers, which consume enormous amount of electrical power [2, 3]. Modern resource-intensive enterprise and scientific applications create increasing demand for high performance computing infrastructures. These large scale computing data centers consume enormous amounts of electrical power because of high availability requirement [4]. This not only has resulted in drastically increased power consumption and power loss, but also has negative impact on our environment. Irrespective of the fact that whether the load is high or low, a minimum number of Server Machines must be always active. This is the first stage of power loss. Losses are actually twofold. Continuous working of a machine surely increases the operating temperature of Physical Machines, and this increased temperature of the Physical Machine affects its reliability, performance, and availability. So, we need to provide some system, which cools the computer facility continuously. This is second stage of drastic power losses. So, it is the biggest need of the hour that the Cloud resources should be utilized very efficiently. This can result in decrease in the size of electrical bill and also heat dissipation up to some extent. In this regard, temperature aware resource management deserves more research in field of Cloud computing. There is a lot of research work that has already been done in field of temperature efficient resource management. Beloglazov and Buyya [2] suggest dynamic thermal management techniques. Researchers in [2] are the first who dealt with the thermal architecture of the data centers. Computing at higher temperatures is more error-prone than computing at moderate temperatures; in fact, component failure rate doubles with every 18 F (or 10°C) increase in temperature, according to the Arrhenius' equation [5], defined by the following formula:

(1) $$\begin{array}{c} k=Ae^{E_{a}/R\times T_{a}}, \end{array}$$

where *k* is the rate constant, *A* is the preexponential factor, *E*_*a*_ is the activation energy, *R* is the gas constant, and *T*_*a*_ is the absolute temperature.

In this paper, our major concentration is on temperature of Physical Machines in a Cloud environment. When workload of a machine is continuously increased then at some point of time, the temperature of this Physical Machine may exceed its threshold temperature, that is, maximum temperature that machine can withstand. This can even completely destroy the essential hardware circuitry of the Physical Machine. So, it is necessary to deal with the rising temperature of the Physical Machines and there must be an efficient and effective strategy of allocation and migration of VMs which considers the temperature of the Physical Machines before making scheduling decisions.

The rest of the paper is organized as follows. In Section 2, current technologies of Cloud computing are discussed.  Section 3 discusses various issues in measuring increase in temperature in a virtualized Cloud environment. In Section 4, research work related to our current work is discussed. This section discusses various methods that are currently being used for resource management in Green Clouds. In Section 5, problem statement is briefly discussed. In Section 6, proposed scheduling strategy is discussed and a VM allocation algorithm is presented.  Section 7 presents the experimental results for the proposed algorithm.  Section 8 presents the conclusion, giving scope of future work.

## 2. Preliminaries

### 2.1. Virtualization

Virtualization is the idea of partitioning or dividing the resources of a single server into multiple segregated VMs. Virtualization technology has been proposed and developed over a relatively long period. The earliest use of VMs was by IBM in 1960, intended to leverage investments in expensive mainframe computers. The idea was to enable multitasking—running multiple applications and processes for different users simultaneously. Recently, owing to the rapid growth in IT infrastructure, multicore processors and a wide variety of hardware, operating systems, and so forth have emerged. In this environment, virtualization has had a resurgence of popularity. Virtualization can provide dramatic benefits for a computing system, including increased utilization, energy saving, rapid deployment, improved maintenance capability, isolation, and encapsulation. Moreover, virtualization enables applications to migrate from one server to another while they are still running, without downtime. There are numerous reasons that virtualization is effective in practical scenarios as mentioned below.

- Server and application consolidation: under virtualization, we can run multiple applications at the same time on the same server, resulting in more efficient utilization of resources and thus saving energy.
- Configurability: virtualization allows dynamic configuration and bundling of resources for a wider variety of applications than could be achieved at the hardware level—different applications require different resources (some requiring more storage, others requiring more computing).
- Increased application availability: VM check pointing and migration allow reduction in downtimes and quick failure recovery from unplanned outages with no interruption in service.
- Improved responsiveness: resource provisioning, monitoring, and maintenance can be automated, and common resources can be cached and reused.

### 2.2. Virtual Machines

A VM is a software implementation of a machine (i.e., a computer) that executes programs like a Physical Machine. This differs from a “process VM,” which is designed to run a single program, such as the Java Runtime Environment (JRE). A “system VM” provides a complete system platform that supports the execution of a complete operating system (OS).

The VM lifecycle has six phases: create, suspend, resume, save, migrate, and destroy. Multiple VMs can run simultaneously in the same physical node. Each VM can have a different OS, and a Virtual Machine Monitor (VMM) is used to control and manage the VMs on a single physical node. A VMM is often referred to as a hypervisor.

### 2.3. Virtualization Platforms

**Figure 1:**
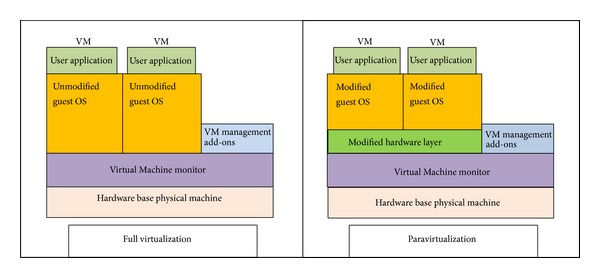
Virtualization platforms.

Virtualization technology has been developed to best utilize computing capacity. In most cases, server virtualization is accomplished by the use of a hypervisor (VMM) to logically assign and separate physical resources. The hypervisor allows a guest operating system, running on the Virtual Machine, to function as if it is solely in control of the hardware, unaware of the fact that other guests are sharing it. Virtualization methods can be classified into two categories according to whether or not the guest OS kernel needs to be modified, as shown in Figure 1. First is full virtualization (supported by VMware (http://www.vmware.com/), Xen (http://www.xen.org/), KVM (http://kvm.qumranet.com), Microsoft Hyper-V (http://www.microsoft.com/hyper-v-server/en/us/default.aspx), etc.), and second is paravirtualization (supported by Xen (http://www.xen.org)). Full virtualization emulates the entire hardware environment by utilizing hardware virtualization support, binary code translation, or binary code rewriting, and thus the guest OS does not need to modify its kernel. In Paravirtualized architecture, OS-level information about the VM can be passed explicitly from the OS to the VMM [7].

## 3. Issues in Measuring Increase in Temperature in a Virtualized Cloud Environment

Server vendors incorporate power measurement hardware, which can monitor the server's power consumption continuously when they are running [8]. Let** T**_**old**_ be the temperature of a node before activation of a new VM and let** T**_**new**_ be temperature of a node after activation of a new VM; then increase in temperature (Δ**T**), due to initiation of a new VM, can be calculated as

(2) $$\begin{array}{c} \Delta T=T_{new}-T_{old}. \end{array}$$

Despite having previous research to leverage the dedicated measurement hardware, determining the energy consumption of a Virtual Machine and increase in temperature of a server machine (Δ**T**) because of activation of a new VM is still a difficult problem for the following reasons.

- The sampling interval of the integrated power meters is generally a second or a tenth of a second [9], while the unit of scheduling Virtual Machines is very short, from a few hundred *μ*s. to a few tens of ms.
- Virtual Machines in a multicore processor system share and compete with other Virtual Machines for system resources. Therefore, the throughput as well as the amount of energy consumed by a Virtual Machine may vary due to characteristics of other Virtual Machines that run concurrently [10].

Consequently, in order to identify the energy consumption of each Virtual Machine and increase in temperature of a node due to start of a new VM (i.e., Δ**T**), which continually and rapidly changes, the hypervisor must equip the resource accounting scheme that can estimate the energy consumption of a Virtual Machine. It is done by observing its various activities as well as the effects from the other Virtual Machines. The number of active cores in a multicore processor heavily affects the processor power consumption. Although each core is a separate and independent execution unit, they share a lot of components such as on-chip caches, buses, and memory controllers. Therefore, the dynamic power of a processor,** P**_**Processor**,_ can be formulated as (3).

**P**_**Shared**_ is the power consumption by the shared components, and** P**_**Core**_ is the power consumption by each core that is executing instructions:

(3) $$\begin{array}{c} \sum P_{Processor}=P_{Shared}+\sum P_{Core}. \end{array}$$

**Figure 2:**
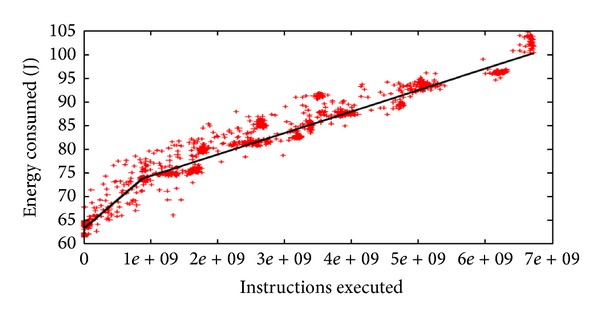
Power consumption depending on a number of *μ*Ops [6].

Consequently, we expect that a processor requires less energy when instructions are spread and are executed over multiple cores than when the same numbers of instructions are executed on a single core or smaller number of cores as shown in Figure 2 [6].

## 4. Related Work

Here, related research work that has been done in field of resource management in Green Clouds taking either load or current temperature as the scheduling criteria is briefly discussed. All these researches have their own pros and cons and act as basis of our proposed research.

Sharma et al. [11] discussed a strategy for dynamic thermal management in data centers. They described a PDC (Programmable Data Centre) architecture in which a CRAC (Computer Room Air Conditioning) and the racks of servers have been used. The function of CRAC is to circulate the cool air between the racks and exhaust the heat from the servers. But CRAC fails at some extent because of unequal distribution of cool air or because of mixing of cool air with hot air. So, they suggested migration of workloads among the servers in order to balance the thermal load across the PDC. They also suggested that cooling the racks that are running below the redline temperature can be avoided.

Ayoub et al. [12] suggested a temperature aware dynamic workload scheduling. They allocate the workload from the hot cores to the cold ones. They provide multitier algorithms. They said that dynamic load balancing does not consider the temperature while distributing the workload. They worked at two levels: core level scheduling and socket level scheduling. They introduced a temperature predictor which uses the band limited property. At the socket level, there is a scheduler which manages the job between sockets. It takes temperature, performance, and fan speed information as an input. The techniques used are spreading and consolidation. At the core level, the scheduler determines the predicted temperature of each core and migrates the job from hot to cold ones.

Song et al. [13] have proposed resource allocation to applications according to their priorities in multiapplication virtualized cluster. The approach requires machine learning to obtain utility functions for the applications and defined application priorities. It does not apply migration of VMs to optimize allocation continuously (the allocation is static). To ensure the QoS, the resources are allocated to applications proportionally according to the application's priorities. Each application can be deployed using several VMs instantiated on different physical nodes. In resource management decisions only CPU and RAM utilizations are taken into account.

Kusic et al. [14] have discussed the problem of power management in virtualized heterogeneous environments using help of the Limited Look-ahead Control (LLC). The objective of authors is to maximize the resource provider's profit by minimizing both power consumption and SLA violation. Kalman filter algorithm is applied to estimate the number of future requests to predict the future state of the system and perform necessary reallocations in the system via reallocation. Liu et al. [15] described an approach which aims to reduce the power consumption in data centers by reducing some of the turned on servers. In order to achieve this, the authors present an architecture composed of some components such as monitoring services, a migration manager, the managed environment, and the front end that provides information to users.

Moore et al. [16] proposed a method for automatic reconfiguration of thermal load management system taking into account thermal performance for improving cooling efficiency and power consumption. They also proposed thermal management solutions focusing on scheduling workloads bearing in mind temperature aware workload placement. Bash and Forman [17] proposed a workload placement strategy for a data center that allocate resources in the areas which are easier to cool resulting in cooling power savings. Ramos et al. [18] proposed a software prediction infrastructure called C-Oracle that makes online estimations for data center thermal management based on load reorganization and DVS. Heath et al. [19] proposed emulation tools for investigating the thermal implications of power management. Liu et al. [20] discussed priority-based consolidation of parallel workloads in Clouds.

## 5. Problem Statement

When workload of a multicore server machine is continuously increased, then, at some point of time, the temperature of this machine may exceed its maximum working temperature, that is, threshold temperature. If a machine runs beyond this maximum temperature, then the risk of hardware or software failure is more and machine requires significant amount of cooling. Excessive heating can even completely destroy the essential hardware circuitry of the Physical Machine. So, it is necessary to proactively deal with the rising temperature of the Physical Machines and there must be an efficient and effective strategy of allocation and migration of VMs which considers the temperature of the Physical Machines before making scheduling decisions, so that maximum threshold temperature is never reached.

## 6. Proposed Prediction Based Proactive Thermal Virtual Machine Scheduling in Green Clouds

### 6.1. Proposed System Design

Proposed design for temperature aware workload scheduling considers current temperature and maximum working temperature, that is, threshold temperature of every machine, before making scheduling decisions. It is the core temperature which is set by original equipment manufacturer. When a machine is running on or beyond threshold temperature, then, to avoid risk of hardware or software failure, significant cooling is required. In this paper, we save the machine from reaching its threshold temperature and save power by reducing the demand of cooling system using a prediction mechanism. The threshold temperature can be the same or different for different Server Machines. Let maximum threshold temperature of a server machine be *T*_Th_ and let current temperature of a server machine be *T*_cu_. *T*_Th_ is the temperature beyond which a machine may not efficiently work and will require excessive cooling to be provided. *T*_cu_ is the temperature on which the machine is currently running in a virtualized Cloud environment. The heuristic chosen for VM scheduling is the difference between threshold and current temperature [21], as formulated in

(4) $$\begin{array}{c} H\;\;=\;\;T_{\text{Th}}-T_{\text{cu}}. \end{array}$$

**Figure 3:**
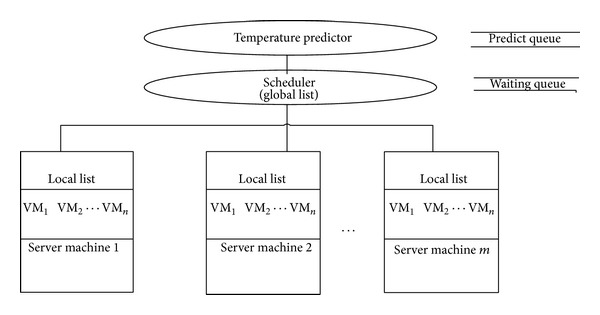
Proposed system design.

Proposed system design for predictive temperature based workload scheduling consists of three levels: Top, Middle, and Bottom [22], as shown in Figure 3.

In top level, a temperature predictor resides, which proactively estimates temperature *T*_n_ of each node. *T*_n_ is the temperature, predicted by temperature predictor for each node, assuming that the application that has arrived is executed on that particular node, which actually is not yet done. Then the predicted temperature is stored in the predict queue. It then finds *T*_Th_ − *T*_n_. The node with maximum value of *T*_Th_ − *T*_n_ is the best candidate for VM allocation. So, we modify the heuristic equation (4) shown above and we have a modified heuristic (**H**_**m**_) as shown in

(5) $$\begin{array}{c} H_{m}\;\;=\;\;T_{\text{Th}}-T_{\text{n}}. \end{array}$$

Scheduler sorts the nodes in decreasing order of **H**_**m**_. Scheduler allocates the VMs to the Physical Machine which is at the first place in the Predict queue because the predicted temperature of this node is farthest away from its threshold temperature.

In middle level, a scheduler resides that allocates VMs to the Physical Machine, which is the best candidate among all machines, that is, whose predicted temperature is farthest away from its maximum threshold temperature. Scheduler keeps track of each Physical Machine in virtualized system and maintains information in a “Unified list” that contains current temperature and threshold temperature of each node. This list is given as input to the temperature predictor for predicting temperature of each node. The Unified list is to be updated after fixed interval of time, say after every 10 ms, by the scheduler. This interval of time would be set by the Cloud administrator, on the basis of type of workload. Besides this fixed interval of time, scheduler will update Unified list when either a new VM request arrives, or migration of VMs occurs, or shutdown of VMs takes place.

Scheduler also manages a waiting queue. It is the queue through which request for new Virtual Machine is entertained. Every new request for Virtual Machine is added at the end of waiting queue. If VM request gets a node, the scheduler will remove that VM request from waiting queue; otherwise it will move further in waiting queue and service the queue till end. Allocation of VMs to Server Machines, according to current temperature, is the main function of the scheduler. This is done in order to keep a server machine's temperature below threshold temperature in a virtualized Cloud environment.

Bottom level consists of Physical Machines on which VMs run. These Physical Machines are connected to the scheduler. Every Physical Machine will maintain a local list which contains the information regarding the VMs, that is, VM name, type of operating system, CPU utilization, and number of processors required.

### 6.2. Prediction Based Proactive Thermal Virtual Machine Scheduling

Temperature Predictive Technique for workload scheduling is presented in Algorithm 1. This algorithm considers happening of five different events.

- *Event (Application_Arrival):* it is an event that is generated when a new application arrives to scheduler for processing. This application is then stored in waiting queue until it gets a VM on some SM.
- *Event (Application_Complete):* it is an event that is generated when an application which was allocated by scheduler for processing to some VM on a SM gets complete. Resources allocated to this application need to be freed up, on occurrence of this event.
- *Event (Free_VM):* It is an event that is generated when an Application Complete event has occured or VM migration needs to be done, for the purpose of server consolidation.
- *Event (Hibernate_SM):* it is an event that is generated when a SM has no more active VMs and can now be hibernated and thus we can have energy saving.
- *Event (Activate_SM):* it is an event that is generated when new application arrives to scheduler for processing, but no SM has capacity to activate a new VM. So, in this case a new SM needs to be activated and the newly arrived application is then allocated to a new VM on this SM.

## 7. Experimental Evaluation

Before implementing the predictive temperature aware resource scheduling technique for Green Clouds, platform needs to be set up, followed by setting Paravirtualized Cloud environment on that platform. Platform is set up using four nodes having configuration as listed in Table 1. The nodes which were available at the time of experimentation were available in pairs, although it is not required to have nodes of the same configuration. These nodes are connected via LAN. FTP is used to transfer VM files, that is, disk image files (.vmdk files) from one node to another.

Implementation and evaluation has been done in virtualized estimation model Xen 4.0. On each node “Core Temp” [23] is installed to get current temperature of that node on the basis of which scheduling of Virtual Machines is to be performed. “Core Temp” makes it easy to monitor the temperature of any modern x86 based processor. This software supports processors from all three major manufacturers: Intel, AMD, and VIA. The temperature readings are very accurate as the data is collected directly from a Digital Thermal Sensor (DTS) which is located in each individual processing core, near the hottest part. This sensor is digital, which means that it does not rely on an external circuit located on the motherboard to report temperature. Its value is stored in a special register in the processor so that software can access and read it. This eliminates any inaccuracies that can be introduced by external motherboard circuits and sensors. The proposed algorithm is applied for evaluation under different scenarios, shown below in Table 2.

In this paper, out of above listed scenarios, results obtained for the first two scenarios are shown below. All the results obtained show efficiency achieved by the proposed algorithm, with respect to existing energy saving techniques.

### 7.1. Scenario 1

In Scenario 1 (S1), four machines having configuration shown in Table 1 were taken. In this scenario all the nodes are made moderately busy. Current and threshold temperatures of these nodes are listed in Table 3. Contents of Unified and Local lists before allocation of new VM request are shown in Tables 3, 4, 5, 6, and 7.

Now, if a new VM request arrives, before allocating it to any of the server machine, predictor will be approached for finding best candidate among all Server Machines for VM allocation. Temperatures predicted by predictor are shown in Table 8.

From the predict table, it can be seen that node 2 is the best candidate for VM allocation, so new VM request is serviced by this node. Contents of Unified and Local list after allocation of Virtual Machines are shown in Tables 9, 10, 11, 12, and 13.

In this scenario, after allocating new Virtual Machine request to node 2, temperature of all the nodes is below threshold temperature. None of the machines requires excessive cooling. This results in decrease in power consumption. It can be seen that temperature of all nodes varies slightly with time, depending on CPU utilization and other factors.

**Figure 4:**
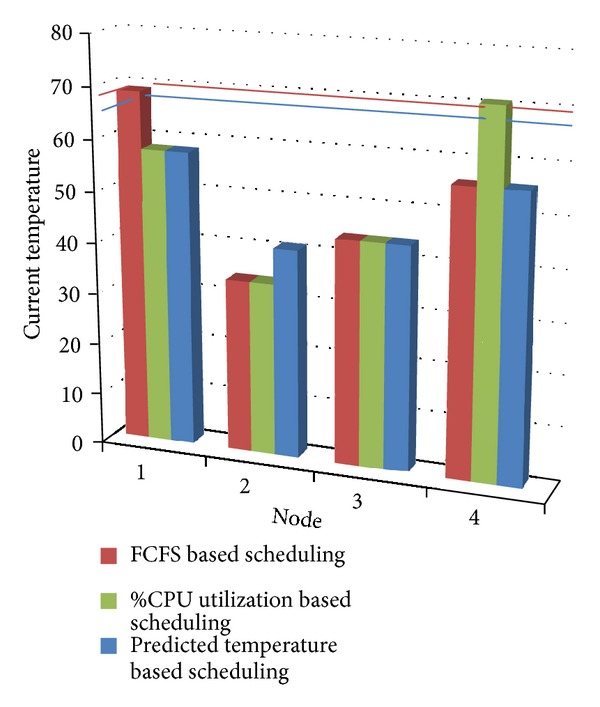
Comparison of FCFS, %CPU utilization, and predicted temperature based scheduling for Scenario 1.

Figure 4 shows the experimental results obtained for Scenario 1, by the proposed algorithm. Also a comparison is made among “First Come First Serve” scheduling method, “%CPU Utilization” scheduling method, and the proposed algorithm in Figure 4. In FCFS method, the first node in the node list is allocated an application if it has capacity to start a new VM; otherwise further nodes in the list are checked, unless a suitable node is found or end of list has been reached; that is, no node is currently available. In “%CPU Utilization” method the nodes are sorted in increasing order of CPU utilization. The node having lowest CPU utilization is given an application, if it has capacity to start a new VM; otherwise further nodes in the list are checked, unless a suitable node is found or end of list has been reached. These scheduling strategies have been compared with our proposed scheduling strategy in Figure 4.

It can be seen from Figure 4 that, in FCFS resource scheduling method, current temperature of node 1 becomes greater than its threshold temperature (65°, shown by blue line in graph) and so requires more cooling. Also it can be seen that in “%CPU Utilization” method temperature of node 4 goes above its threshold temperature (68°, shown by red line in graph). With our proposed method, temperature of none of the nodes exceeds their threshold temperature, so cooling requirement is also less as compared with other methods.

### 7.2. Scenario 2

In Scenario 2 (S2), one of the nodes taken is highly busy, second node is completely idle, and the rest of the nodes are moderately busy, as can be seen from Table 2. To start with, five applications are made running on node 1, node 2 is completely idle (has just become idle), and node 3 and node 4 each have two applications running. The contents of Unified and Local list, before allocation of Virtual Machine to new application, are shown in Tables 14, 15, 16, 17, and 18. It can be seen from Table 14 that most heavily loaded node, that is, node 1, has maximum current temperature.

Now, if a new application arrives, scheduler schedules it on the basis of predictions made by the predictor. The algorithm first checks number of active VMs on each node and finds that node 2 has no active VM (as shown by *). Hence, node 2 is hibernated rather than assigning any new application. Now, the updated Unified list is given to predictor which predicts temperature *T*_n_ of each node, for the new application. Predicted temperatures are shown in Table 19. From this table it is found that node 3 is the best candidate for VM allocation as its predicted temperature is farthest away from its threshold temperature. After allocation of a new Virtual Machine, the Unified and Local lists are shown in Tables 20, 21, 22, and 23.

**Figure 5:**
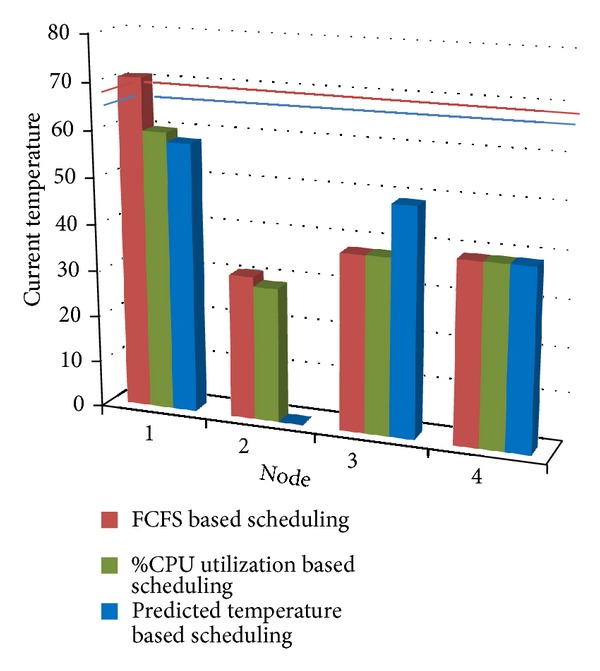
Comparison of FCFS, %CPU Utilization, and predicted temperature based scheduling for Scenario 2.

In this case, new VM is started on node 3. This helps node 1 and node 4 to keep their temperature below threshold temperature. Also node 2 is hibernated, which further saves energy.  Figure 5 shows the experimental results obtained for Scenario 2, by the proposed algorithm. Also a comparison is made among “First Come First Serve” scheduling method, “%CPU Utilization” scheduling method, and the proposed method in Figure 5.

It can be seen from Figure 5 that, in FCFS resource scheduling method, current temperature of node 1 again becomes greater than its threshold temperature (65°_,_ shown by blue line) and so requires more cooling. Also it can be seen that, in “%CPU Utilization” method, because %CPU utilization of node 2 is minimum, next job is allocated to it rather than switching it off. With our proposed method, node 2 is hibernated and also temperature of none of the nodes is allowed to exceed their threshold temperature. So, cooling requirements are also less as compared with other methods.

**Figure 6:**
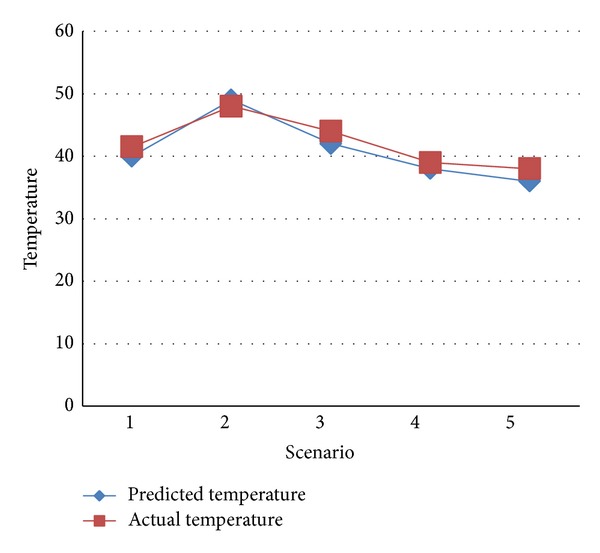
Comparison of actual and predicted temperature for Scenarios 1–5.

It can be seen from Tables 8, 11, 19, and 22 that there can be some difference between actual and predicted temperature of a node to which incoming application is assigned. The difference between actual and predicted temperature for all the scenarios is shown in Figure 6. The less the difference between actual and predicted temperature, the better the efficiency of the system.

## 8. Conclusion and Scope of Future Work

As the prevalence of computing still continues to rise, the need for power saving mechanisms and reducing CO_2_ footprints is increasing. In this paper, we have presented a prediction based green scheduling algorithm, on the basis of current temperature and threshold temperature of the nodes for improving system efficiency. Here we have found new ways to save vast amounts of energy while minimally impacting performance. Analysis of the results shows that this algorithm can effectively improve the average resource utilization of the system and reduce energy consumption. Future opportunities could explore a scheduling system that is both power aware and thermal aware simultaneously, to maximize energy savings both from physical servers and the cooling systems used. Such a scheduler would also drive the need for better data centre designs, both in server placements within racks and closed-loop cooling systems integrated into each rack. Also, temperature predictions of “Temperature Predictor” can be made self-learning and autonomic, based on feedback given by all the nodes, regarding their actual operating temperature after allocation of new application to it, to the temperature predictor [24].

## Figures and Tables

**Algorithm 1:**
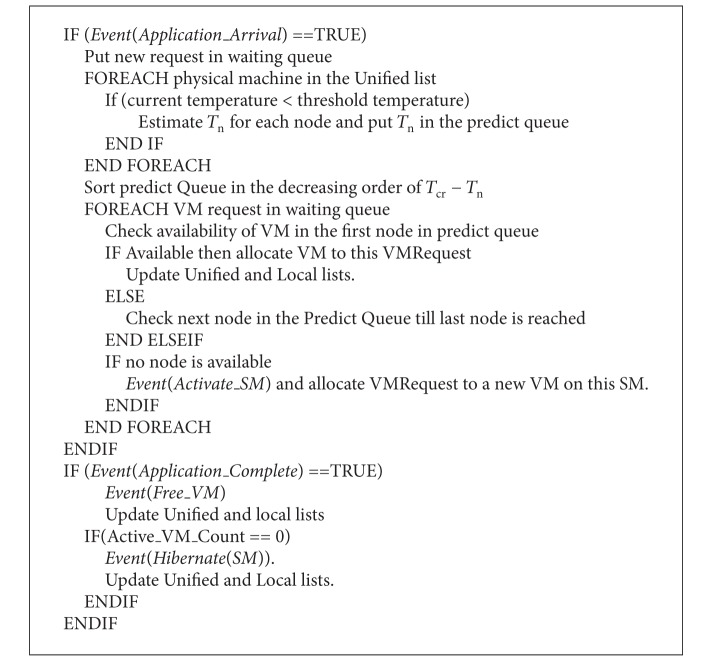
Proactive thermal VM scheduling.

**Table 1:** Hardware configuration.

Type	Specification1 (node 1 and node 2)	Specification2 (node 3 and node 4)
Processor	Intel Core i7-3612QM	Intel Core i7-860
CPU speed	2.1 GHz	2.8 GHz
Memory	2 GB	4 GHz
Operating system	Windows 7, 32 bits	Linux
Number of cores	04	04

**Table 2:** Workload scenarios.

	Load on node 1	Load on node 2	Load on node 3	Load on node 4
Scenario 1	Moderate	Moderate	Moderate	Moderate
Scenario 2	High	Idle	Moderate	Moderate
Scenario 3	High	Idle	Idle	Moderate
Scenario 4	High	Moderate	Moderate	Moderate
Scenario 5	High	High	High	Idle

**Table 3:** Unified list (S1).

Node ID	Current temperature (°C)	Threshold temperature (°C)
1	58	65
2	34	65
3	44	68
4	56	68

**Table 4:** Local list of node 1 (S1).

VM name	Operating system	CPU utilization (%)	Number of processors	RAM (MB)
VM1	RHEl 5	16	1	126
VM2	RHEl 6	19	1	512
VM3	Windows XP	25	2	128

**Table 5:** Local list of node 2 (S1).

VM name	Operating system	CPU utilization (%)	Number of processors	RAM (MB)
VM1	RHEl 6	18	1	128
VM2	Windows XP	25	1	128

**Table 6:** Local list of node 3 (S1).

VM name	Operating system	CPU utilization (%)	Number of processors	RAM (MB)
VM1	RHEL 6	18	1	128
VM2	Windows XP	15	1	128
VM3	Windows XP	35	1	256

**Table 7:** Local list of node 4 (S1).

VM name	Operating system	CPU utilization (%)	Number of processors	RAM (MB)
VM1	RHEL 6	15	1	256
VM2	Windows XP	10	1	128
VM3	Windows XP	11	1	128

**Table 8:** Predict table (S1).

Node ID	Threshold temperature (*T*_Th_)	Predicted temperature (*T*_n_)	*H*_*m*_ = *T*_Th_ − *T*_n_
1	65	62	03
2	65	40	25
3	68	51	17
4	68	63	05

**Table 9:** Unified list (S1).

Node ID	Current temperature (°C)	Threshold temperature (°C)
1	58	65
2	41.5	65
3	44.5	68
4	56.5	68

**Table 10:** Local list of node 1 (S1).

VM name	Operating system	CPU utilization (%)	Number of processors	RAM (MB)
VM1	RHEl 5	16	1	126
VM2	RHEl 6	19	1	512
VM3	Windows XP	25	2	128

**Table 11:** Local list of node 2 (S1).

VM name	Operating system	CPU utilization (%)	Number of processors	RAM (MB)
VM1	RHEl 6	18	1	128
VM2	Windows XP	25	1	128
**VM3**	**RHEL 5**	**20**	**1**	**256**

**Table 12:** Local list of node 3 (S1).

VM name	Operating system	CPU utilization (%)	Number of processors	RAM (MB)
VM1	RHEL 6	18	1	128
VM2	Windows XP	18	1	128
VM3	Windows XP	35	1	256

**Table 13:** Local list of node 4 (S1).

VM name	Operating system	CPU utilization (%)	Number of processors	RAM (MB)
VM1	RHEL 6	16	1	256
VM2	Windows XP	10	1	128
VM3	Windows XP	11	1	128

**Table 14:** Unified list (S2).

Node ID	Current temperature (°C)	Threshold temperature (°C)
1	60	65
2	21	65
3	38	68
4	39	68

**Table 15:** Local list of node 1 (S2).

VM name	Operating system	CPU utilization (%)	Number of processors	RAM
VM1	RHEl 5	15	1	126
VM2	RHEl 6	18	1	512
VM3	RHEL 6	16.2	2	256
VM4	Windows XP	24	1	136
VM5	Windows XP	12	2	128

**Table 16:** Local list of node 2 (S2).

VM name	Operating system	CPU utilization (%)	Number of processors	RAM
∗				

*Represents absence of any active VM.

**Table 17:** Local list of node 3 (S2).

VM name	Operating system	CPU utilization (%)	Number of processors	RAM
VM1	RHEl 5	15	1	126
VM2	RHEl 6	18	1	512

**Table 18:** Local list of node 4 (S2).

VM name	Operating system	CPU utilization (%)	Number of processors	RAM
VM1	RHEl 5	20	1	126
VM2	RHEl 6	16	1	512

**Table 19:** Predict table (S2).

Node ID	Threshold temperature (*T*_Th_)	Predicted temperature (*T*_n_)	*H*_*m*_ = *T*_Th_ − *T*_n_
1	65	70	−05
3	68	49	19
4	68	50	18

**Table 20:** Unified list (S2).

Node ID	Current temperature (°C)	Threshold temperature (°C)
1	60	65
3	48	68
4	39	68

**Table 21:** Local list of node 1 (S2).

VM name	Operating system	CPU utilization (%)	Number of processing cores	RAM (MB)
VM1	RHEl 5	14	1	128
VM2	RHEl 6	16	1	512
VM3	RHEL 6	15.1	2	256
VM4	Windows XP	26	1	136
VM5	Windows XP	20	2	128

**Table 22:** Local list of node 3 (S2).

VM name	Operating system	CPU utilization (%)	Number of processing cores	RAM (MB)
VM1	RHEl 5	15	1	126
VM2	RHEl 6	18	1	512
**VM3**	**RHEL 5**	**15**	**1**	**256**

**Table 23:** Local list of node 4 (S2).

VM name	Operating system	CPU utilization (%)	Number of processing cores	RAM (MB)
VM1	RHEl 5	20	1	126
VM2	RHEl 6	16	1	512
